# Higher Purity of Phosphatidylserine Improves Human Cortical Neuron Function by Modulating SIRT1-PGC-1α Pathways

**DOI:** 10.3390/brainsci16020194

**Published:** 2026-02-06

**Authors:** Sung-Min Jeon, Stanley Cho, Yoon-Seob Lee, Ji-Yu Lee, Eunice J. Kang, Tommy D. Kim, Jayna Shin, Heejin Jo, Sung-Ung Kang

**Affiliations:** 1 Neuroregeneration and Stem Cell Programs, Institute for Cell Engineering, School of Medicine, Johns Hopkins University, Baltimore, MD 21205, USA; 2 Department of Chemical and Biomolecular Engineering, Cornell University, Ithaca, NY 14853, USA; 3 Department of Dentistry, College of Dentistry, Yonsei University, Seoul 03722, Republic of Korea; 4 The Yonsei Dental Clinic, Suwon 16385, Republic of Korea; 5 Department of Korean Medicine, School of Medicine, CHA University, Seoul 06062, Republic of Korea; 6 Department of Neurology, School of Medicine, Johns Hopkins University, Baltimore, MD 21205, USA

**Keywords:** phosphatidylserine (PS), PS purity, aging, Aβ_42_-induced Alzheimer’s disease model, brain health, mitochondrial quality control, SIRT1-PGC-1α axis

## Abstract

While phosphatidylserine (PS) is recognized for its neuroprotective properties, the effects of PS purity on human cortical neurons remain unexplored. This study investigates the effects of three different PS purities (15 µM of 50%, 70%, and 80%) on neuronal health using human-embryonic-stem-cell-derived cortical neurons. Our findings reveal that higher PS purity enhances the expression of key regulatory proteins Sirtuin 1 (SIRT1) and Peroxisome proliferator-activated receptor gamma coactivator 1-alpha (PGC-1α), known for their roles in neuroprotection and mitochondrial function. Specifically, 80% PS purity significantly increases SIRT1 and PGC-1α levels, suggesting that PS purity strengthens neuroprotective pathways and improves mitochondrial quality control. Through SIRT1 knockdown experiments, we demonstrate that PS-induced upregulation of PGC-1α is SIRT1 dependent, highlighting a SIRT1-PGC-1α regulatory axis that enhances mitochondrial health. In an amyloid-beta 1–42 (Aβ_42_)-induced Alzheimer’s disease (AD) model, PS treatment reduced cytotoxicity and countered the Aβ_42_-induced downregulation of SIRT1 and PGC-1α, particularly at 70% and 80% PS purity, indicating PS’s role in preserving neuronal viability and combating AD-like pathology. These results suggest that the biological activity of PS preparations in vitro can depend on purity, motivating future studies to define compositional determinants and bioavailability relevant to translational applications.

## 1. Introduction

Phosphatidylserine (PS) is a crucial phospholipid that plays an essential role in maintaining brain function [1,2]. Predominantly located in the inner leaflet of cell membranes, PS contributes to various neuronal processes, including signal transduction, synaptic transmission, and neuroplasticity [3,4]. PS is abundant in the brain, comprising 13–15% of the total phospholipid content in the cerebral cortex, where it supports neuronal membrane fluidity and the functional integrity of membrane-bound proteins, including receptors and ion channels [5,6]. The binding of PS to key proteins on the cell membrane facilitates the assembly of signaling complexes critical for synaptic transmission and plasticity [7,8]. Recent studies have highlighted the neuroprotective effects of PS, particularly in enhancing cognitive functions such as memory, attention, and processing speed [9,10]. Clinical trials suggest its potential as a dietary supplement for improving cognition, especially in aging populations and patients with early stages of neurodegenerative disorders, such as Alzheimer’s disease (AD) and other types of dementia, making it a candidate for addressing age-related cognitive decline [11,12]. However, commercial PS preparations used as nutraceutical ingredients can differ widely in PS content and formulation, and systematic comparisons testing whether ‘purity grade’ influences PS-driven signaling responses in human cortical neurons remain limited.

Emerging evidence suggests that PS supplementation can modulate the expression of key transcriptional coactivators such as peroxisome proliferator-activated receptor gamma coactivator 1-alpha (PGC-1α) [13,14]. PGC-1α is a master regulator of mitochondrial biogenesis and energy metabolism, enhancing mitochondrial function in response to various physiological stimuli [15,16]. In the brain, PGC-1α supports energy metabolism, antioxidant defense, and synaptic function. PGC-1α is important in neuroprotection, including upregulation of antioxidant enzymes, such as superoxide dismutase (SOD) and catalase, which mitigate damage from reactive oxygen species (ROS) [17,18]. PGC-1α also regulates the neurotrophic factors essential for synaptic health and neuroplasticity [19,20]. Dysregulation of PGC-1α is linked to neurodegenerative diseases such as Parkinson’s disease (PD) and AD, where mitochondrial dysfunction and oxidative stress are prevalent [21,22]. Understanding how PS affects PGC-1α expression in neurons could provide valuable insights into potential neuroprotective strategies [14]. Additionally, Sirtuin 1 (SIRT1), a protein deacetylase that plays a critical role in cellular aging, metabolic regulation, and neuroprotection, has emerged as a potential modulator of PGC-1α [23,24]. SIRT1 can activate PGC-1α by deacetylation, enhancing its activity in mitochondrial function, oxidative stress reduction, and neuroprotection [25,26]. Given that both SIRT1 and PGC-1α are involved in neuroprotective pathways, investigating how PS affects their expression could uncover important mechanisms underlying PS’s potential benefits in neurodegenerative conditions [27,28].

In this study, we examined the effect of PS on both SIRT1 and PGC-1α expression using human embryonic stem cell (hESC)-derived cortical neurons. hESC-derived neurons serve as a reliable model for studying neuronal physiology and disease mechanisms, closely mimicking human cortical neuron properties. By using this model, we aimed to investigate whether the purity of PS impacts its ability to modulate SIRT1 and PGC-1α expression. The purity of supplements can significantly influence their efficacy and safety, as impurities may affect bioavailability, alter pharmacodynamics. Thus, investigating PS purity is essential for optimizing neuroprotective strategies, ensuring consistent therapeutic outcomes, and advancing the use of PS in combination with other supplements or therapeutic agents in neurodegenerative disease management.

## 2. Materials and Methods

### 2.1. Preparation of Phosphatidylserine (PS)

Powdered PS of 80%, 70%, and 50% purity was purchased from the 80% PS (Notified ingredient No. 2–29, manufactured by KONO CHEM Co., Ltd, CO, USA), and 70% and 50% PS (Notified ingredient No. 2–29, CERTIFIED NUTRACEUTICALS, Inc., CA, USA). PS was prepared following standard lipid handling procedures [29,30]. Briefly, PS was first dissolved in chloroform/methanol in a glass vial under a chemical fume hood to generate a concentrated stock solution. The solvent was evaporated under a gentle stream of nitrogen gas to form a uniform lipid film, followed by vacuum desiccation to ensure complete removal of residual organic solvent. The dried lipid film was then rehydrated in sterile, 0.22-µm-filtered PBS and dispersed via brief sonication or membrane extrusion to generate small unilamellar vesicles. PS concentration was calculated based on molecular weight, and the suspension was diluted into culture medium to a final concentration of 15 µM immediately before neuronal cell treatment. Unless otherwise indicated, neurons were treated with PS for 16 h and subsequently replaced with fresh culture medium for 32 h prior to harvest for immunoblotting and qPCR. For Aβ_42_ experiments, PS was applied together with Aβ_42_ and refreshed with each medium change during the 7-day exposure period.

### 2.2. Differentiation of Human ESCs into Cortical Neurons

H1 human embryonic stem cells (hESCs) were differentiated toward a cortical neuronal fate following a published protocol [31]. Briefly, H1 hESC colonies were released from mouse embryonic fibroblast feeder layers and cultured as aggregates in suspension using human ESC medium lacking FGF2 for 6 days in low-attachment six-well plates (Thermo Fisher Scientific Inc., MA, USA). On day 7, embryoid bodies (EBs) were transferred onto Matrigel-coated plates to promote attachment and the emergence of neuroepithelial rosette-like structures. Rosette-containing aggregates (RONAs) were manually isolated to generate neurospheres, maintained for 24 h, dissociated into single cells, and plated onto laminin/poly-D-lysine–coated plates for downstream experiments. For neuronal maturation/differentiation, cells were maintained in neural differentiation medium consisting of Neurobasal supplemented with B27 (Invitrogen, MA, USA) and the following additives: brain-derived neurotrophic factor (BDNF; 20 ng/mL; PeproTech, MA, USA), glial-cell-line-derived neurotrophic factor (GDNF; 20 ng/mL; PeproTech, MA, USA), ascorbic acid (0.2 mM; Sigma, MO, USA), dibutyryl adenosine 3′5′-monophosphate (cAMP; 0.5 mM; Sigma, MO, USA). Patterning factors were applied as indicated: retinoic acid (2 μM), SHH (50 ng/mL), purmorphamine (2 μM), or a combination of retinoic acid + SHH + purmorphamine. 

### 2.3. Amyloid-β1–42 Preparation and Treatment

Synthetic human amyloid-β1–42 (Aβ_42_) peptide was prepared using established oligomer-enrichment protocols [32,33]. Briefly, lyophilized Aβ_42_ (rPeptide, GA, USA) was monomerized and aliquoted according to published procedures, reconstituted in DMSO, and diluted into PBS to generate working stocks. Preparations were incubated under defined conditions to promote oligomer formation and used immediately or stored as aliquots at −80 °C. hESC-derived cortical neurons were exposed to 10 μM Aβ_42_ for 7 days [31,34,35,36], with medium changes every (48–72 h); PS was added 30 min prior to Aβ_42_ treatment. Neuronal death was quantified by Hoechst 33342/propidium iodide staining as described.

### 2.4. Cell Death and Viability Assessment

hESC-derived cortical neurons were exposed to 10 μM Aβ_42_ for 7 days. To assess cell death, nuclei of all cells were labeled with Hoechst 33342 (7 μM), while membrane-compromised (non-viable) cells were identified via propidium iodide (PI) staining (2 μM; Invitrogen, USA). Fluorescence images were acquired using a Zeiss microscope, and the total number of nuclei and PI-positive cells were quantified using Axiovision software version 4.8 (Carl Zeiss, OR, USA). Cell viability was independently evaluated using the Alamar Blue assay (Invitrogen, MA, USA), with fluorescence measured at an excitation wavelength of 570 nm and an emission wavelength of 585 nm according to the manufacturer’s instructions.

### 2.5. Luciferase Reporter Assays

Transcriptional activity of the human Pgc-1α promoter was assessed using a dual-secreted luciferase reporter system (Secrete-Pair Dual Luminescence Assay, GeneCopoeia, MD, USA). hESC-derived neurons were transfected with a lentiviral construct harboring a 1.3 kb fragment of the human Pgc-1α promoter (−1274 to +26 relative to the transcription start site) upstream of a Gaussia luciferase reporter, along with a constitutively expressed secreted alkaline phosphatase (SEAP) control cassette. Three days after transfection, Neurons were transfected with a SIRT1 expression vector and treated with phosphatidylserine (PS) as indicated for 48 h. Culture supernatants were collected for luminescence measurements. Gaussia luciferase activity was quantified by incubating 10 μL of conditioned medium with 100 μL of Gaussia luciferase substrate buffer for 30 s. To measure SEAP activity, aliquots of supernatant (10 μL) were heat inactivated at 65 °C for 5 min and subsequently incubated with SEAP substrate for an additional 5 min. Gaussia luciferase signals were normalized to corresponding SEAP values to control for variations in transduction efficiency.

### 2.6. Quantitative PCR

Total RNAs from cultured human cortical neurons were extracted using a RNeasy mini kit (Qiagen Sciences, Inc., MD, USA) and quantified using a UV–Vis spectrophotometer (NanoDrop 2000, Thermo Fisher Scientific Inc., MA, USA). RNA was reverse transcribed using a high capacity cDNA reverse transcription kit (Applied Biosystems, MA, USA). cDNAs were amplified with PowerUp™ SYBR Green Master Mix (Applied Biosystems, MA, USA) on a StepOnePlus™ system (Applied Biosystems, MA, USA). The amounts of cDNA within each sample were normalized to 18sRNA or glyceraldehyde 3-phosphate dehydrogenase (GAPDH). Sequences for primer set were used: Human Drp1: (F) 5′-TTTGCTCGTGTGAAGACTGG-3′ and (R) 5′-TCCTGGAGCTTCCTTTCTGA-3′; Human Fis1: (F) 5′-CTACAGGGGTGCAGGAGAAA-3′ and (R) 5′-AGATG GACTGGTAGGCATGG-3′; Human Mfn1: (F) 5′-CAGAGAAGAGGGTTTATTCA-3′ and (R) 5′-ACTCATCAACCAAAACAGAT-3′, Mfn2: (F) 5′-TGAATGTTGTGTTCTTTCTG -3′ and (R) 5′-AAGTGCTCTCTGCTAAATGT-3′ [37].

### 2.7. Immunoblot Assay

Protein lysates obtained from cultured human cortical neurons were separated on 4–20% gradient SDS–PAGE and subsequently transferred onto nitrocellulose membranes. Membranes were blocked in PBS containing 0.05% (*v*/*v*) Tween-20 and 5% (*w*/*v*) bovine serum albumin to prevent nonspecific binding. Following blocking, membranes were incubated with primary antibodies targeting SIRT1 (rabbit polyclonal, #2310, Cell Signaling Technology, MA, USA), PGC-1α (rabbit polyclonal, NBP1-04676, Novus Biologicals, CO, USA), MAP2 (rabbit polyclonal, #4542, Cell Signaling Technology, MA, USA), VGLUT1 (mouse monoclonal, N28/9, Abcam, MA, USA), Synapsin I (rabbit monoclonal, ab254349, Abcam, MA, USA), TBR1 (rabbit monoclonal, ab183032, Abcam, MA, USA), SATB1 (rabbit monoclonal, ab92446, Abcam, MA, USA), and β-actin (HRP-conjugated rabbit monoclonal, 13E5, Cell Signaling Technology, MA, USA). After washing three times, membranes were incubated with horseradish peroxidase-linked secondary antibodies against rabbit or mouse IgG (Amersham Biosciences, NJ, USA). Immunoreactive bands were visualized using enhanced chemiluminescence reagents (Thermo Scientific, USA) and captured on X-ray film (Amersham Bioscience Corp., USA). Band intensities were quantified using ImageJ software version 1.54r, and densitometric values represent the mean of minimum three independent experiments. Two different housekeeping controls, β-actin and GAPDH, were used for the immunoblot and qRT-PCR, respectively, because it showed stable band intensity across our neuronal treatment conditions and displayed stable Ct values across conditions in our neuronal cultures.

### 2.8. Statistics

Quantitative data are presented as the mean ± SEM. Statistical significance was assessed either via an unpaired two-tailed Student’s *t*-test or an ANOVA test with Tukey’s HSD post hoc analysis for comparison of more than three groups.

## 3. Results

This study investigated the effects of PS purity on neuronal function using hESC-derived cortical neurons, examining PS at purities of 50%, 70%, and 80%. Additionally, the role of PS in mitigating AD-like pathology was assessed using an Aβ_42_ peptide model. The findings demonstrate how PS purity influences critical protein expression, mitochondrial quality control, and neuroprotection.

### 3.1. Effects of PS Purity on SIRT1 and PGC-1α Expression in hESC-Derived Cortical Neurons

**Figure 1:**
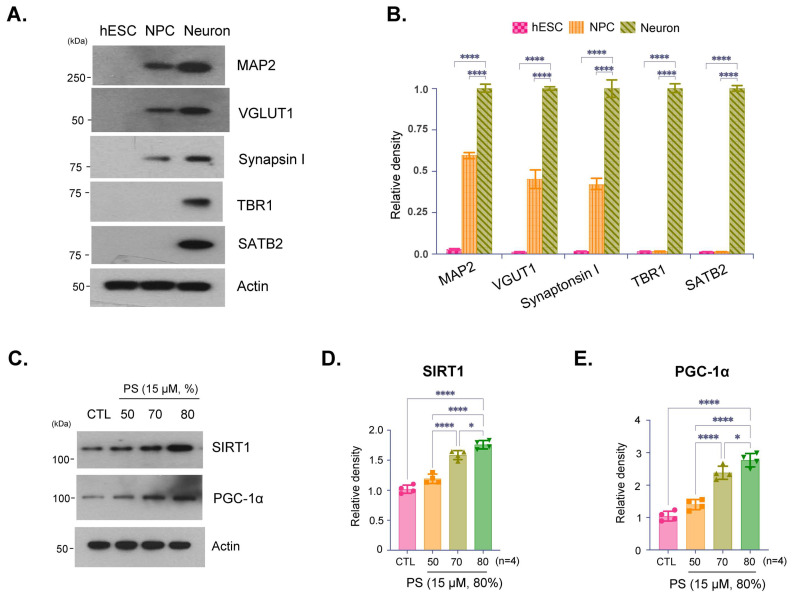
Effects of Phosphatidylserine (PS) purity on SIRT1 and PGC-1α expression in hESC-derived cortical neurons. (**A**) Immunoblot analysis of cultured hESC-derived cortical neurons showed expression of MAP1, VGLUT, Synapsin I, TBR1, and SATB2, confirming that both cortical identity and synaptic integrity were preserved in the neurons. (**B**) Quantification of relative protein density from Immunoblots normalized to actin. (**C**) Immunoblot analysis of SIRT1 and PGC-1α protein expression in hESC-derived cortical neurons treated with PS (15 µM) at different purities (50%, 70%, and 80%) and a control (CTL). (**D**,**E**) Quantification of relative SIRT1 (**D**) and PGC-1α (**E**) protein density from Immunoblots normalized to actin. Data are presented as means ± SEM, with *n* = 4 biological replicates. Statistical significance is indicated as follows: * *p* < 0.05, **** *p* < 0.0001.

Immunoblot analysis confirmed the developmental stage specific characters and neuronal identity of the cultured hESC-derived cortical neurons (Figure 1A,B). Expressed protein levels of immunoblotting of the synaptic markers vesicular glumatate transporter (VGLUT) and Synapsin I indicate that synaptic integrity was preserved (Figure 1A,B). Furthermore, expression of microtubule-associated protein 2 (MAP2), T-box brain protein 1 (TBR1), and special AT-rich sequence-binding protein 2 (SATB2) confirmed the cortical identity and differentiation of these neurons, indicating that they are functionally active and suitable for further analysis (Figure 1A,B). Notably, Figure 1A is presented as a qualitative characterization panel to confirm neuronal identity, cortical layer-marker expression, and synaptic marker presence in our hESC-derived cortical neuron cultures. These blots are not intended for quantitative comparison across markers and conditions, and quantitative densitometry is therefore provided only for the defined experimental comparisons shown in Figure 1B–E. Immunoblot analysis showed expression of SIRT1 and PGC-1α, proteins involved in cellular aging and metabolic regulation, in neurons treated with PS at 50%, 70%, and 80% purity (Figure 1C). The results indicate a purity-dependent increase in SIRT1 and PGC-1α expression with higher PS purities, suggesting that increased PS purity promotes the expression of proteins. Quantification of SIRT1 and PGC-1α expression levels showed a statistically significant increase in SIRT1 and PGC-1α with higher PS purity levels, particularly at 80% (Figure 1D,E).

### 3.2. SIRT1-Dependent Regulation of PGC-1α Expression by PS in hESC-Derived Cortical Neurons

**Figure 2:**
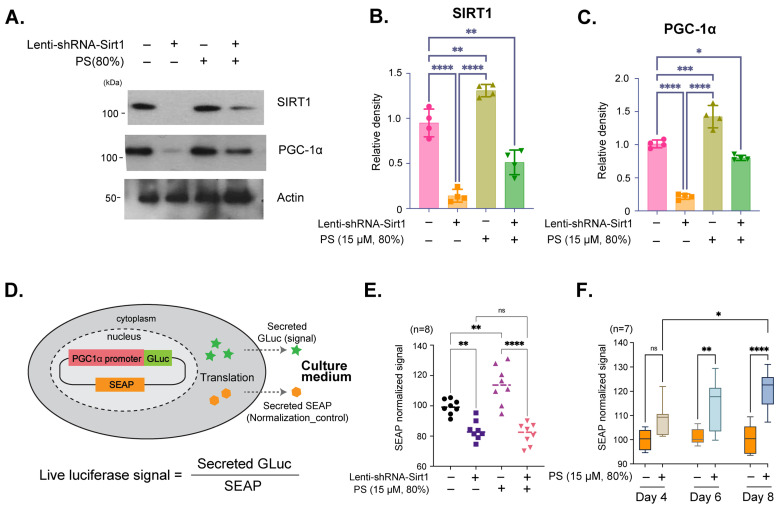
SIRT1-dependent regulation of Pgc-1α expression by PS (15 µM, 80%) in hESC-derived cortical neurons. (**A**) Immunoblot images of SIRT1 and PGC-1α protein expression in hESC-derived cortical neurons. Cells were treated with Lenti-shRNA-Sirt1 to knock down SIRT1 expression and subsequently treated with 80% PS purity (15 µM). (**B**,**C**) Quantification of relative SIRT1 (**B**) and PGC-1α (**C**) protein density, normalized to actin. (**D**) Schematic illustration of the Secrete-Pair™ Dual Luminescence Assay used to measure *PGC-1α* promoter activity. The *PGC-1α* promoter drives expression of Gaussia Luciferase (GLuc) as a signal reporter, while Secreted Alkaline Phosphatase (SEAP) is used as a normalization control. (**E**) Quantification of *PGC-1α* promoter activity in hESC-derived cortical neurons after treatment with Lenti-shRNA-Sirt1 and 80% purity PS (15 µM). The GLuc signal, normalized by SEAP, shows Sirt1-dependent PGC-1α promoter activity. (**F**) Time-dependent measurement of Pgc-1α promoter activity following PS treatment (15 µM, 80%) at Day 4, 6 and 8. Data are presented as means ± SEM, with sample sizes as indicated in each panel. Statistical significance is indicated as follows: * *p* < 0.05, ** *p* < 0.01, *** *p* < 0.001, **** *p* < 0.0001.

Immunoblot analysis showed the effects of SIRT1 knockdown on PGC-1α expression in neurons treated with 80% PS purity. Lenti-shRNA-Sirt1 was used to reduce SIRT1 expression, followed by treatment with 80% PS purity (Figure 2A). The results showed a decrease in PGC-1α expression with SIRT1 knockdown, which is partially restored with PS treatment, indicating that PS mediates PGC-1α expression through SIRT1 (Figure 2A–C). Quantification of SIRT1 and PGC-1α protein demonstrated that SIRT1 expression is significantly reduced with Lenti-shRNA-SIRT1, and PS treatment could restore SIRT1 levels to some extent (Figure 2B). Additionally, PGC-1α expression was shown to be SIRT1-dependent, as indicated by the significant decrease in its levels upon SIRT1 knockdown, partially recovered with PS treatment (Figure 2C). The Secrete-Pair™ Dual Luminescence Assay was employed to measure live *PGC-1α* promoter activity without sacrificing cells and to observe changes in activity over time (Figure 2D). The *PGC-1α* promoter (1.3 kb from TSS) drives the expression of Gaussia Luciferase (GLuc) as a signal reporter, with Secreted Alkaline Phosphatase (SEAP) acting as a normalization control for assay accuracy as illustrated in Figure 2D. The assay results showed the normalized GLuc signal for neurons treated with Lenti-shRNA-SIRT1 and 80% PS purity, indicating that *PGC-1α* promoter activity is SIRT1-dependent and upregulated with PS treatment (Figure 2E). Additionally, the effects of treatment with PS (80%) on Day 4, 6, and 8 were evaluated to observe changes in Pgc-1α promoter activity. The time-dependent increase in *Pgc-1α* promoter activity demonstrated sustained activation over time (Figure 2F).

### 3.3. Protective Effects of PS Purity on SIRT1 and PGC-1α Expression in an AD Model

**Figure 3:**
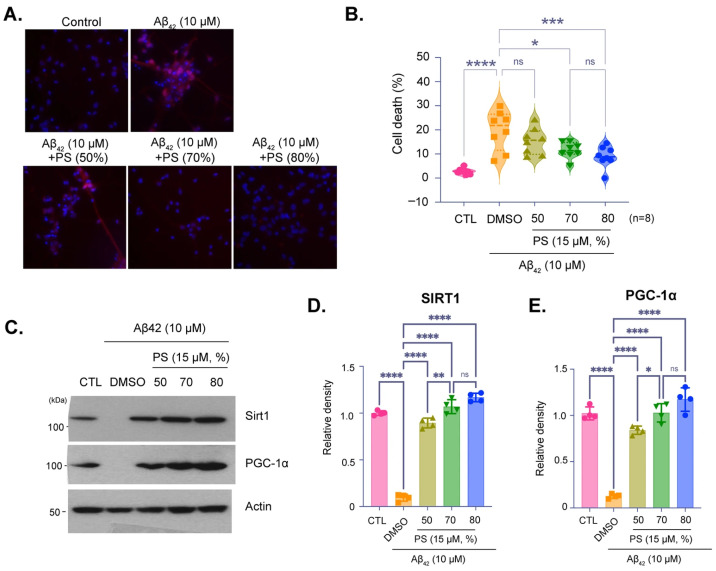
Protective effects of PS purity on SIRT1 and PGC-1α expression in an Alzheimer’s Disease (AD) Model. (**A**) Representative images of hESC-derived cortical neurons treated with Aβ_42_ peptide (10 µM) to induce AD-like conditions, followed by treatment with PS at varying purities (50%, 70%, and 80%). Cells were stained with propidium iodide (PI) to assess cell viability, with PI-positive cells indicating cell death. (**B**) Quantification of cell death percentage based on PI staining. (**C**) Immunoblot analysis of SIRT1 and PGC-1α protein expression under Aβ_42_ treatment, with and without PS at different purities. (**D**,**E**) Quantification of relative SIRT1 (**D**) and PGC-1α (**E**) protein density from immunoblot, normalized to actin. Data are presented as means ± SEM, with sample sizes as indicated in each panel. Statistical significance is indicated as follows: ns—non-significant, * *p* < 0.05, ** *p* < 0.01, *** *p* < 0.001, **** *p* < 0.0001.

Representative images and quantification of cell viability in hESC-derived cortical neurons treated with Aβ_42_ peptide (10 µM) to simulate AD-like pathology, with or without PS at varying purities (Figure 3A). Images show propidium iodide (PI) staining for cell death, with PI-positive cells indicating cytotoxicity (Figure 3A). Quantification showed that PS treatment significantly reduces Aβ_42_-induced cell death, with 80% purity providing the most effective neuroprotection (Figure 3B). Immunoblot analysis of SIRT1 and PGC-1α expression in neurons treated with Aβ_42_ and PS at different purities (Figure 3C–E). Aβ_42_ treatment reduced the expression of these proteins however, PS treatment, particularly at both 70% and 80% purities, mitigated this reduction, suggesting that PS helps preserve critical proteins associated with cellular health. Quantitative analysis of SIRT1 and PGC-1α levels normalized to actin showed that treatment of Aβ_42_ significantly reduces SIRT1 and PGC-1α expression, while PS treatment, especially at 70% and 80%, restores their levels, demonstrating its protective effect against AD-like pathology (Figure 3D,E).

### 3.4. PS-Induced Mitochondrial Quality Control via SIRT1 and PGC-1α in an AD Model

**Figure 4:**
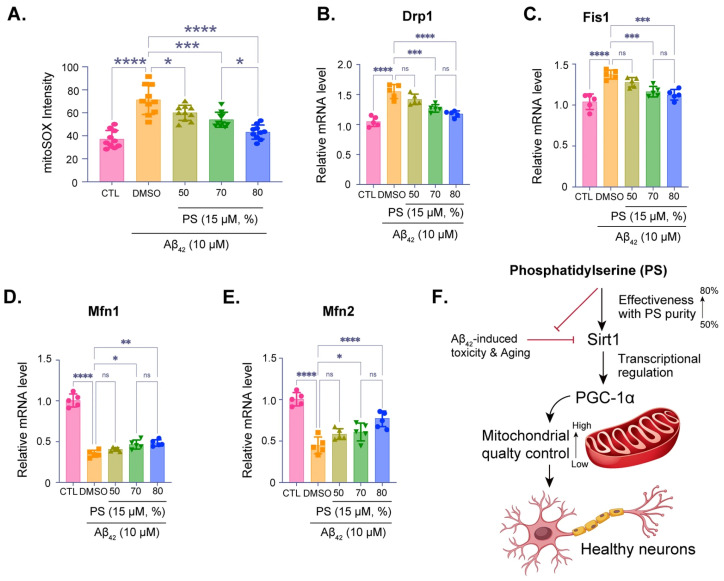
Phosphatidylserine-Induced mitochondrial quality control via SIRT1 and PGC-1α in an AD model. (**A**) MitoSOX assay quantifying mitochondrial oxidative stress by measuring superoxide levels in hESC-derived cortical neurons exposed to Aβ_42_ (10 µM) with varying levels of PS purity (50%, 70%, 80%). (**B**–**E**) qPCR analysis of Drp1 (**B**) and Fis1 (**C**) mRNA levels, genes associated with mitochondrial fission, Mfn1 (**D**) and Mfn2 (**E**) mRNA levels, genes associated with mitochondrial fusion. (**F**) Summary diagram illustrating the proposed mechanism: PS at higher purities mitigates Aβ_42_-induced toxicity and oxidative stress by enhancing SIRT1 expression, which in turn regulates PGC-1α. This pathway promotes mitochondrial quality control, supporting healthy neuronal function. Data are presented as means ± SEM, with sample sizes as indicated in each panel. Statistical significance is indicated as follows: ns—non-significant, * *p* < 0.05, ** *p* < 0.01, *** *p* < 0.001, **** *p* < 0.0001.

Measurement of mitochondrial superoxide levels using mitoSOX staining, showing oxidative stress in neurons treated with Aβ_42_ and various purities of PS (Figure 4A). Higher PS purity significantly reduced oxidative stress, as indicated by the decrease in mitoSOX intensity, with 80% purity showing the most effective reduction (Figure 4A). qPCR analysis of the mitochondrial fission-related genes *Drp1* (Figure 4B) and *Fis1* (Figure 4C), both of which are upregulated under Aβ_42_-treatment, and mitochondrial fusion-related genes *Mfn1* (Figure 4D) and *Mfn2* (Figure 4E), both of which are downregulated under the Aβ_42_-treatment, suggesting mitochondrial dysfunction, were quantitatively investigated with various purities of PS. PS treatment, particularly at 70% and 80% purities, restores the mitochondrial superoxide levels, supporting mitochondrial fission and fusion, and its integrity stress (Figure 4B–E). Summary diagram of the proposed mechanism, indicating that PS at higher purities (up to 80%) mitigates Aβ_42_-induced toxicity and oxidative stress by enhancing SIRT1 expression, which regulates PGC-1α (Figure 4F). This pathway promotes mitochondrial quality control and sustains neuronal health.

## 4. Discussion

This study highlights the potential of PS in preserving neuronal health through purity-dependent modulation of key regulatory proteins, specifically, SIRT1 and PGC-1α, using hESC-derived cortical neurons. The Rosette Neural Aggregate (RONA) method is known to efficiently mimic the human cortex by differentiating hESCs into a functionally mature neuron complex using timed retinoic acid application (Figure 1A,B) [3,31,38]. This culture allows to effectively replicate human cortical architecture and reliable testing of chemical efficacy and neurotoxicity in a controlled environment [3,31,38,39]. By integrating this system, our data underscores the importance of PS purity in neuronal function, showing that higher PS purity enhances neuroprotective pathways, attenuates AD-like pathology, and supports mitochondrial quality control, providing insights into how PS purity could influence neurodegenerative disease progression. Consistent with prior studies demonstrating that SIRT1 plays a critical role in cellular aging, metabolic regulation, and neuroprotection, we observed a significant, purity-dependent increase in SIRT1 expression [40,41,42]. Specifically, 80% PS purity resulted in the highest levels of SIRT1 and PGC-1α, a transcriptional coactivator regulated by SIRT1 that potentially enhances mitochondrial biogenesis and function (Figure 1C–E). This finding aligns with previous research suggesting that SIRT1-mediated upregulation of PGC-1α can contribute to neuroprotection by promoting mitochondrial health and reducing oxidative stress [40,42,43]. This effect of PS purity suggests that PS enhances the SIRT1-PGC-1α axis, potentially mitigating age-related declines in these proteins in cortical neurons. Notably, neurons can display spatially regulated PS externalization under physiological conditions. Scott-Hewitt and colleagues reported that exposed PS at synaptic membranes functions as an ‘eat-me’ cue during developmental microglia-mediated synaptic pruning, and masking extracellular PS with Annexin V partially reduced synapse elimination [44]. These findings suggest further experiments to directly track exogenous PS incorporation and to determine whether PS supplementation alters leaflet distribution or local PS exposure dynamics in human cortical neurons. Moreover, even when the nominal PS concentration is held constant, differences in the accompanying non-PS lipid fractions or excipients across purity grades could alter vesicle or aggregate properties and thereby modulate effective cellular PS delivery and uptake, which can substantially influence membrane PS levels and downstream responses [45,46].

To further investigate the regulatory relationship between SIRT1 and PGC-1α, we conducted experiments using SIRT1 knockdown, revealing that PS-induced PGC-1α expression is indeed SIRT1-dependent (Figure 2A–C). This SIRT1-mediated modulation of PGC-1α is critical, as PGC-1α is known for its role in mitochondrial quality control, which is essential in high-energy-demanding cells such as neurons [41,42,43]. Our Secrete-Pair™ Dual Luminescence Assay, used to measure *PGC-1α* activity, demonstrated that PS treatment activates the *PGC-1α* promoter in a SIRT1-dependent manner, supporting previous findings that SIRT1 can regulate PGC-1α transcriptionally (Figure 2D–F) [41,42,43]. This observation provides evidence that PS exerts its effects on mitochondrial health through a SIRT1-PGC-1α regulatory axis, emphasizing the relevance of PS purity in therapeutic strategies or supplements targeting mitochondrial dysfunction [40,42,47]. Related research by Hao Li and colleagues [48] in a neuronal context reported that activating stress-response metabolic pathways such as AMPK activation using resveratrol promotes mitochondrial biogenesis and reduces oxidative damage in SH-SY-5Y cells and in an in vivo prenatal stress paradigm [48]. While the upstream trigger differs from our PS paradigm, these findings support the broader concept that mitochondrial quality control programs in neuronal cells are tunable and context dependent. We chose 15 μM PS as a single working concentration based on prior studies demonstrating that exogenous PS analogs can be loaded into mammalian cells in the ~10 μM range to probe PS-dependent membrane biology without overt toxicity [49,50], and on evidence that PS-containing liposomes can increase cellular PS content in neural cells in a dose- and time-dependent manner [45].

The neuroprotective effects of PS were further substantiated in an Aβ_42_-induced AD model, where we observed that PS, particularly at 70% and 80% purities, mitigated Aβ_42_-induced cytotoxicity, as demonstrated by reduced cell death (Figure 3A,B). These findings corroborate prior studies suggesting that SIRT1 and PGC-1α play protective roles against AD pathology [41,43,51]. Notably, treatment with higher than 70% PS purity counteracted the Aβ_42_-induced downregulation of SIRT1 and PGC-1α, indicating that PS not only preserves neuronal viability but supports the expression of essential proteins in the face of neurotoxic insults (Figure 3C–E). This suggests that PS supplementation, particularly at high purities, may serve as a viable approach to mitigate early AD pathology by modulating critical proteins involved in cellular health [41,47,52]. Although we focused on representative mitochondrial dynamics markers such as Drp1,Fis1, Mfn1, and Mfn2 because altered fission–fusion balance is strongly implicated in AD-related mitochondrial dysfunction (Figure 4A–E) [53,54]. As PGC-1α also promotes mitochondrial biogenesis via NRF-dependent programs [55] further studies can be considered to quantify biogenesis endpoints such as NRF1, TFAM, and mtDNA content to complement the dynamics readouts. Because PS is typically administered orally as a supplement, its in vivo efficacy will depend on digestion/absorption and formulation-dependent bioavailability; therefore, the present study should be interpreted as a controlled in vitro assessment of direct neuronal responses independent of ADME variables.

Given the observed effects on mitochondrial regulators, we explored the impact of PS on mitochondrial quality control in the Aβ_42_-treated neurons. MitoSOX staining revealed a significant reduction in mitochondrial oxidative stress with increasing PS purity, with the highest purity (80%) showing the most pronounced effect (Figure 4A). Additionally, our qPCR data indicated that PS treatment modulated the expression of mitochondrial fission (*Drp1* and *Fis1*) and fusion (*Mfn1* and *Mfn2*) genes, which are often dysregulated in —conditions, including AD (Figure 4B–E) [42,53,54]. Previous studies suggest that mitochondrial dynamics are critical for maintaining mitochondrial health and neuronal survival, and the observed restoration of fission–fusion gene expression with high-purity PS points to its role in promoting mitochondrial stability and function [41,42,53]. Our findings thus extend the current understanding of PS’s neuroprotective role in the human cortical neuron model, proposing that PS purity is a key determinant in modulating mitochondrial homeostasis under AD-like conditions (Figure 4F).

## 5. Conclusions

This study suggests that PS purity may influence neuroprotective efficacy in hESC-derived cortical neurons, with higher-purity preparations showing a tendency toward stronger activation of the SIRT1–PGC-1α axis and improved mitochondrial quality control. PS increased SIRT1 and promoted PGC-1α promoter activity in a SIRT1-dependent manner, as SIRT1 knockdown abolished PS-induced PGC1α upregulation. In an Aβ_42_-induced AD-like model, PS, particularly at higher nominal purities, was associated with reduced cytotoxicity, partial normalization of mitochondrial fission/fusion gene expression, and lower oxidative stress. Importantly, this conclusion must be interpreted with the study’s limitation: the 80% PS preparation was sourced from a different manufacturer than the 50% and 70% PS, so supplier-specific formulation or impurity profiles could contribute to the apparent purity-dependent effects.

## 6. Limitation

A key limitation of this study is the potential confounding between nominal PS purity and manufacturer specific production variables. Although we tested PS preparations labeled as 50%, 70%, and 80% purity, the 50% and 70% PS was produced using the same company’s manufacturing process, whereas the 80% PS was sourced from a different manufacturer. Therefore, it would be inappropriate to conclude that “higher purity universally produces superior neuroprotective function,” because the observed differences, especially those involving the 80% condition, could partially reflect supplier-dependent factors such as distinct impurity profiles, fatty acid composition, oxidation status, or differences in accompanying non-PS lipid fractions that alter vesicle or aggregate properties and effective cellular delivery and uptake.

The most consistent and pronounced separation was observed between 50% and 70% PS (same production pipeline), supporting the possibility that PS content itself contributes to the directionality of the effects in at least part of the dataset. Nonetheless, definitive attribution of the strongest responses to purity alone requires additional validation. The most direct way to strengthen causal interpretation would be to (i) use analytically characterized PS preparations spanning multiple purities from a single source, coupled with compositional profiling (e.g., LC–MS/HPLC) to quantify non-PS fractions and impurities, and (ii) directly track exogenous PS incorporation and membrane distribution including leaflet localization and local PS exposure dynamics, e.g., via labeled PS and Annexin V-based assays. In addition, broader mitochondrial endpoints (NRF1, TFAM, mtDNA content) and dose- and time-response analyses would help further confirm whether the SIRT1-PGC1α linked effects generalize across conditions and are not driven by formulation-specific bioavailability in vitro.

## Data Availability

The original contributions presented in this study are included in the article. Further inquiries can be directed to the corresponding authors.
